# European Rabbits as Reservoir for *Coxiella burnetii*

**DOI:** 10.3201/eid2106.141537

**Published:** 2015-06

**Authors:** David González-Barrio, Elisa Maio, Madalena Vieira-Pinto, Francisco Ruiz-Fons

**Affiliations:** Spanish Wildlife Research Institute, Ciudad Real, Spain (D. González-Barrio, F. Ruiz-Fons);; University of Trás-os-Montes e Alto Douro, Vila Real, Portugal (E. Maio, M. Vieira-Pinto)

**Keywords:** *Coxiella burnetii*, bacteria, lagomorph, Q fever, European rabbits, Oryctolagus cuniculus, reservoir, wildlife, zoonoses, Iberian region, Europe

## Abstract

We studied the role of European rabbits (*Oryctolagus cuniculus*) as a reservoir for *Coxiella burnetii* in the Iberian region. High individual and population seroprevalences observed in wild and farmed rabbits, evidence of systemic infections, and vaginal shedding support the reservoir role of the European rabbit for *C. burnetii*.

Wildlife play a major role in the maintenance and transmission of multihost pathogens (*1**,**2*). Understanding the role of host species involved in multihost zoonotic pathogen maintenance and transmission is essential to prevent disease caused by these pathogens.

*Coxiella burnetii*, which is the cause of Q fever, is a zoonotic pathogen that infects multiple hosts (*3*). The implication of wildlife in the life cycle of *C. burnetii* has been reported worldwide (*4**,**5*), and wildlife might act as a source for humans infections (*6**,**7*).

European rabbits (*Oryctolagus cuniculus*) are native to the Iberian Peninsula and have been introduced into Australia, New Zealand, Chile, and Argentina (*8*). Domestic varieties of European rabbits are farmed worldwide. Specific ecologic traits (high density, gregarious behavior, high reproductive rate) suggest that these rabbits might become a major reservoir of zoonotic pathogens. However, whether *C*. *burnetii* can infect, replicate in, and be shed by European rabbits and contaminate the environment is not known. In this study, we investigated the role of these rabbits in a region to which Q fever is endemic.

## The Study

**Figure 1:**
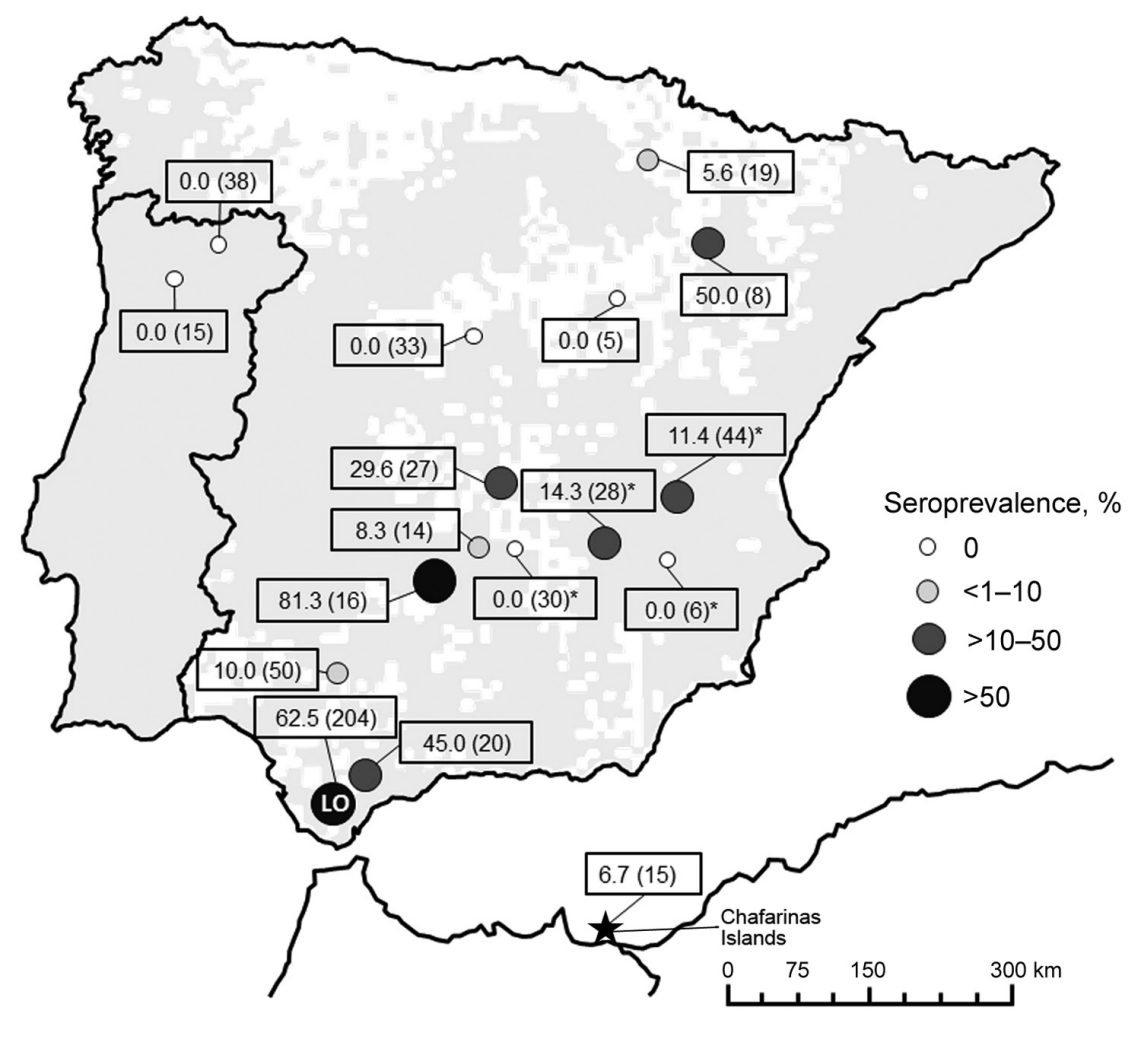
Seroprevalence of *Coxiella burnetii* (sample size) in wild and farmed European rabbits (*Oryctolagus cuniculus*), Iberian Peninsula and Chafarinas Islands. The distribution area of wild rabbits in the Iberian Peninsula (10 x 10 km Universal Transverse Mercator squares) is shown (gray shading) according to Mitchel-Jones et al. (*9*). LO sampling location is indicated. *Rabbit farm.

Serum samples were collected from European wild rabbits in 13 locations in Spain, Portugal, and the Chafarinas Islands during 2003–2013 (Figure 1). Wild rabbits from 1 of the study locations (LO; Figure 1) were obtained from 2 epidemiologic scenarios (*10*). The first scenario involved rabbits that coexisted with farmed red deer (*Cervus elaphus*) (sites A and B). The second scenario involved rabbits that had not been in contact with ruminants since 2002 (site C). 

In addition to serum samples, spleen, uterus, and mammary gland samples and vaginal and uterus swab specimens were collected from rabbits surveyed at location LO. Each rabbit from this location was weighed and sexed. Serum samples were also collected from farmed rabbits on 4 farms in Spain (Figure 1). Samples were stored at −20°C until tested.

Serum samples were analyzed by using the LSIVet Ruminant Q Fever Serum/Milk ELISA Kit (Life Technologies, Carlsbad, CA, USA) and horseradish peroxidase–conjugated protein G (Sigma-Aldrich, St. Louis, MO, USA) as secondary antibody (*10*). Results were interpreted according to manufacturer’s recommendations.

DNA from tissues and swab specimens was extracted bu using the DNeasy Blood and Tissue Kit (QIAGEN, Hilden, Germany). Swabs were incubated at 56°C for 30 min in 200 μL of AL buffer containing 20 μL of proteinase K. Swabs specimens were then vortexed for 15 s and removed. The remaining solution was incubated at 56°C for 30 min. The manufacturer’s blood extraction protocol was then used. DNA aliquots were frozen at −20°C. Negative controls (nuclease-free water; Promega, Madison, WI, USA) were included during DNA extraction.

DNA samples were analyzed by using a conventional PCR (*11*). PCR products were visualized by electrophoresis in 1.2% agarose gels containing 0.1 μL/mL of GelRed Nucleic Acid Gel Stain (Biotium, Hayward, CA, USA).

Logistic regression models were used to test the effect of potential factors (Table) on the individual risk of exposure to *C. burnetii*. Individual ELISA results were included as response variables in the models and the location origin of rabbits was used as a random factor.

**Table:** Variables considered as potential risk factors and outputs (coefficient/statistic) of best fitted risk factor models for *Coxiella burnetii* exposure in European rabbits (*Oryctolagus cuniculus*), Iberian Peninsula and Chafarinas Islands*

Variable code	Variable, units	Cb_sp_	Cb_spLO_	Cb_splLO_	Cb_rtLO_
Intercept	NA	67.776/4.98†	–037270.00‡	–2,942.687/1.15‡	2,925.025/0.49‡
X	Longitude, decimal degrees	§	¶	¶	¶
Y	Latitude, decimal degrees	§	¶	§	¶
Ye	Year	−0.033/0.20‡	§	1.464/0.42‡	−1.453/0.45‡
Se	Season	§	§	§	§
Sp	Spring	1.209/5.45‡	§	−1.583/2.78‡	§
Su	Summer	2.257/5.45†	§	Referent	§
Au	Autumn	0.043/5.45‡	§	§	§
Wi	Winter	Referent	§	§	§
Mg	Management, wild vs. farmed	§	¶	¶	¶
Rum	Ruminants, presence vs. absence	¶	§	0.059/0.0‡	2.004/1.08‡
Sex	Sex, M vs. F	¶	§	−0.383/0.27‡	2.004/0.22‡
Wg	Weight, g	¶	§	§	§

*Cb_sp_,overall seropositivity; Cb_spLO_, seropositivity at location LO; Cb_splLO_, systemic infection (*C. burnetii* DNA in spleen of wild rabbits); CB_rtLO_, shedding (*C. burnetii* DNA in reproductive tracts of wild rabbits); NA, not applicable. †p<0.05. ‡p>0.05. §Variable was included in each model but was not retained in the best model. ¶Variable not tested.

Logistic regression models were also used for individual exposure of rabbits from location LO to *C. burnetii* (ELISA), for the presence/absence of *C. burnetii* DNA in spleen (a proxy of systemic infection), and for the presence/absence of *C. burnetii* DNA in the reproductive tract (a proxy of shedding; including PCR results from uterus, and vaginal and uterus swab specimens). Location LO was included as a random factor, and sex, weight and ruminant presence/absence were also included as predictor variables (Table). Models were created by using a forward stepwise procedure. The model with the lowest Akaike information criterion (*12*) was selected.

Statistical analyses were performed in SPSS version 20.0 (IBM, Armonk, NY, USA). Prevalence-associated, Clopper-Pearson exact 95% CIs were estimated.

Serum samples from 572 rabbits (464 wild and 108 farmed) (Figure 1) were analyzed. Overall seroprevalence was 32.3% (95% CI 28.5%–36.4%) for wild and farmed rabbits, 37.9% (95% CI 33.5%–42.5%) for wild rabbits, and 8.3% (95% CI 3.8%–15.2) for farmed rabbits. Seroprevalence in wild rabbit populations ranged from 6.7% to 81.3%. Nine (64.3%) of 13 wild rabbit populations and 2 (50%) of 4 farms had >1 seropositive rabbit. The best model for *C. burnetii* exposure retained sampling year and season, and the risk for seropositivity was higher in summer (Table).

**Figure 2:**
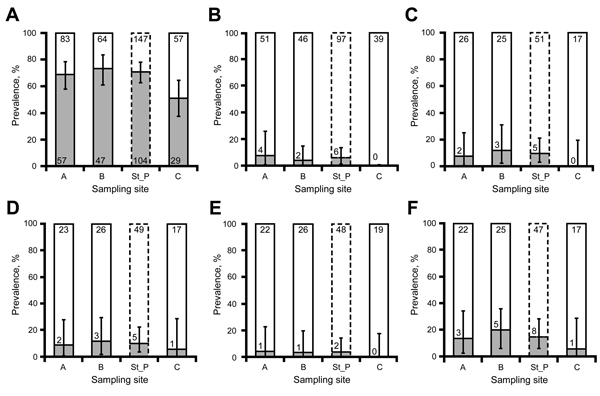
Prevalence of antibodies against *Coxiella burnetii* and *C. burnetii* DNA in European rabbits (*Oryctolagus cuniculus*) at sampling location LO, Iberian Peninsula. A) Antibodies; B) DNA in spleen; C) DNA in vaginal swab specimen; D) DNA in uterine swab specimen; E) DNA in uterus; F) DNA in reproductive tract (vaginal swab specimen, uterine swab specimen, uterus). Gray bars indicate seroprevalence. St_P indicates results for sites with ruminants (sites A and B); no ruminants were present at site C. Values at the top of bars indicate number of samples, and values at the bottom of bars indicate number of positive samples. Error bars indicate prevalence-associated exact 95% CIs.

Seroprevalence at location LO was 65.2% (133/204; 95% CI 58.2%–71.7%); it was slightly lower at site C than at sites A and B (Figure 2, panel A). However, none of the considered factors were retained in the best model (Table). Six (4.4%; 95% CI 1.6%–9.4%) of 136 spleen samples analyzed at location LO were positive by PCR (4 male and 2 female rabbits). Five of the 6 spleen PCR–positive animals were seropositive. The 2 female rabbits were positive for *C. burnetii* DNA in vaginal swab specimens. Spleen PCR–positive rabbits were observed only at sites A and B (Figure 2, panel B).

The best model for the presence of *C. burnetii* DNA in spleen retained sampling year, season, presence of ruminants, and sex (Table). Results suggest expected higher systemic infection prevalence in rabbits coexisting with farmed red deer (Figure 2, panel B). *C*. *burnetii* DNA was detected in the reproductive tract of 9 (14.1%; 95% CI 6.6%–25.0%) of 64 female rabbits at sites A, B, and C (Figure 2, panel F). The presence of ruminants was retained in the best model for *C. burnetii* DNA in the reproductive tract (Table). None of the 13 mammary glands analyzed was positive for *C. burnetii* DNA.

## Conclusions

This study provides 3 results that suggest that European rabbits might be reservoirs of *C. burnetii*. These 3 results are high seroprevalence of this bacteria; systemic infections; and bacterial shedding in vaginal secretions, which, in other host species, constitutes the main source for environmental contamination and transmission between species (*13*).

Host density is a major factor in *C. burnetii* prevalence in livestock (*14*). The highest seroprevalence values were observed at 2 locations where rabbit populations are managed for hunting purposes, which promotes high densities of rabbits. These findings suggest that rabbit density may be a major factor in the ecology of *C. burnetii*. In addition, the European rabbit is a gregarious species with a high reproductive rate. This rate favors transmission of *C. burnetii* from infected to susceptible animals, which is enhanced by replacement of *C. burnetii*–negative rabbits and can contribute further to spread of this bacterium in the environment.

The higher risk of exposure to *C. burnetii* observed during the summer might be related to increased indirect interaction with *C. burnetii* shed by coexisting ruminants, whose main shedding season is late spring–early summer (*3*). Inclusion of ruminants in the final models for systemic infection and vaginal shedding at location LO might support this hypothesis. However, further analyses, including molecular typing of circulating strains, would be needed to confirm the direction, frequency, and magnitude of interspecies interactions favoring transmission of *C. burnetii*.

Indirect transmission of *C. burnetii* between rabbits, humans, livestock, and other wild species may be enhanced in regions with high-density rabbit populations and in regions in which the European rabbit is a major game or farm species. Hunters, game keepers, rabbit farmers, veterinarians, wildlife researchers, livestock producers and livestock might be exposed to *C. burnetii* from rabbits (*6**,**15*). The European rabbit shows a high potential as a reservoir of *C. burnetii* for infection of livestock and humans in Europe.
